# Hot and cold cognitive disturbances in antidepressant-free patients with major depressive disorder: a NeuroPharm study

**DOI:** 10.1017/S0033291720000938

**Published:** 2021-10

**Authors:** V. H. Dam, D. S. Stenbæk, K. Köhler-Forsberg, C. Ip, B. Ozenne, B. J. Sahakian, G. M. Knudsen, M. B. Jørgensen, V. G. Frokjaer

**Affiliations:** 1 Neurobiology Research Unit, the Neuroscience Centre, Copenhagen University Hospital Rigshospitalet, Denmark; 2 Faculty of Health and Medical Sciences, University of Copenhagen, Denmark; 3 Psychiatric Center Copenhagen, Copenhagen University Hospital Rigshospitalet, Denmark; 4 Department of Clinical Pharmacology, H. Lundbeck A/S, Valby, Denmark; 5 Department of Public Health, Section of Biostatistics, University of Copenhagen, Denmark; 6 Department of Psychiatry, University of Cambridge, Cambridge, UK; 7 Behavioral and Clinical Neuroscience Institute, University of Cambridge, Cambridge, UK

**Keywords:** Affective biases, cognitive profiles, cold cognition, EMOTICOM, hot cognition, major depressive disorder, social cognition

## Abstract

**Background:**

Cognitive disturbances are common and disabling features of major depressive disorder (MDD). Previous studies provide limited insight into the co-occurrence of hot (emotion-dependent) and cold (emotion-independent) cognitive disturbances in MDD. Therefore, we here map both hot and cold cognition in depressed patients compared to healthy individuals.

**Methods:**

We collected neuropsychological data from 92 antidepressant-free MDD patients and 103 healthy controls. All participants completed a comprehensive neuropsychological test battery assessing hot cognition including emotion processing, affective verbal memory and social cognition as well as cold cognition including verbal and working memory and reaction time.

**Results:**

The depressed patients showed small to moderate negative affective biases on emotion processing outcomes, moderate increases in ratings of guilt and shame and moderate deficits in verbal and working memory as well as moderately slowed reaction time compared to healthy controls. We observed no correlations between individual cognitive tasks and depression severity in the depressed patients. Lastly, an exploratory cluster analysis suggested the presence of three cognitive profiles in MDD: one characterised predominantly by disturbed hot cognitive functions, one characterised predominantly by disturbed cold cognitive functions and one characterised by global impairment across all cognitive domains. Notably, the three cognitive profiles differed in depression severity.

**Conclusion:**

We identified a pattern of small to moderate disturbances in both hot and cold cognition in MDD. While none of the individual cognitive outcomes mapped onto depression severity, cognitive profile clusters did. Overall cognition-based stratification tools may be useful in precision medicine approaches to MDD.

## Introduction

Disturbance of cognitive functioning is a common feature of major depressive disorder (MDD) and has been proposed as an important treatment target (Collins et al., 2011). Cognitive symptoms including inability to concentrate or difficulty making decisions are listed among the diagnostic criteria for MDD (APA, 2013; WHO, 2007). Investigations into cognitive disturbances in MDD have typically focused on either so-called ‘hot’ or ‘cold’ cognitive functions (Roiser & Sahakian, 2013). Hot cognition describes mental functions that involve the processing of emotionally salient information (e.g. identifying emotional facial expressions) or emotional responses (e.g. reward-driven behaviours). In particular negative affective biases, i.e. the subconscious allocation of more attention and mental resources to the processing of negative information over positive information, have been associated with MDD psychopathology (Elliott, Zahn, Deakin, & Anderson, 2011; Miskowiak & Carvalho, 2014) and may play a key role in the onset and maintenance of depressive symptoms (Roiser, Elliott, & Sahakian, 2012). Another hot cognitive domain which may be impaired in MDD is social cognition which includes functions such as seeing oneself in the ‘other’, i.e. Theory of Mind (Bora & Berk, 2015; Wolkenstein, Schonenberg, Schirm, & Hautzinger, 2011), interpretation of social situations and excessive experiences of negative social emotions such as shame and guilt (Kim et al., 2015).

Cold cognition describes mental processes that include emotionally neutral information and do not directly involve activation of emotional states (Roiser & Sahakian, 2013). Recent meta-analyses suggest that cold cognitive deficits in MDD are predominantly found in domains of attention, learning and memory and executive functions (Goodall et al., 2018; Lee, Hermens, Porter, & Redoblado-Hodge, 2012; Rock, Roiser, Riedel, & Blackwell, 2014). Slowed reaction time has also been reported for depressed patients and is, along with agitated psychomotor function, considered a distinct symptom in MDD (Bennabi, Vandel, Papaxanthis, Pozzo, & Haffen, 2013).

The effects sizes reported for cognitive disturbances in MDD are typically small to moderate which is relatively modest compared to those reported for other serious neuropsychiatric disorders such as Alzheimer's Disease and schizophrenia (Maruff & Jaeger, 2016). Nevertheless, their impact on daily life may be very disruptive. Both hot and cold cognitive disturbances in MDD have been found to be detrimental to the patient's ability to engage successfully in work or educational activities as well as overall psychosocial functioning (Cambridge, Knight, Mills, & Baune, 2018; Weightman, Knight, & Baune, 2019). This is especially relevant as cognitive disturbances do not always resolve with the remission of core depressive symptoms (Hernaus, Gold, Waltz, & Frank, 2018).

Despite a growing body of data on specific cognitive deficits in MDD, we currently know little about the co-occurrence and magnitude of impairments across different types of cognitive domains. Few studies on MDD have included both hot and cold cognitive tasks and comparisons between studies are often hampered by differences in cohort characteristics such as medication/treatment status, comorbidity, age-range, chronicity and severity of current depressive episode. To address this, we therefore applied a broad range of both hot and cold cognitive tasks in a large cohort of well-characterised and antidepressant-free depressed patients.

## Methods

### Participants and study design

One hundred non-psychotic antidepressant-free patients suffering from a moderate to severe depressive episode lasting less than two years [Hamilton Depression Rating Scale-17 (HDRS_17_) ⩾18] were included in a large multimodal neuroimaging clinical trial (NeuroPharm 1). Patients were eligible for inclusion if they had been antidepressant free for >2 months; had not previously exhibited non-response to SSRIs; and had not undergone more than one antidepressant treatment attempt in the current depressive episode. Patients were recruited through their primary care centre or a central referral site for ‘depression treatment packages’ at the Mental Health Services of the Capital Region of Copenhagen. MDD diagnosis was confirmed by a certified psychiatrist and corroborated by a Mini-International Neuropsychiatric Interview (MINI). Out of the 100 patients who entered the study, neuropsychological data was available from 92 patients (67 females); out of these, 41 patients had first-episode depression while 51 patients had recurrent depression. In addition, data from 100 healthy participants were collected as part of a validation study of the EMOTICOM test battery, a novel neuropsychological battery specifically designed to assess hot cognitive functions (Dam et al., 2019), and additionally three healthy controls were recruited via internet advertisements and flyers posted around the greater Copenhagen area (52 females). Exclusion criteria for the study were history of psychiatric disorders for healthy controls and prior or present history of other primary axis I psychiatric disorders for depressed patients; significant somatic illness, brain trauma; use of psychotropic medication within 4 weeks of inclusion; significant lifetime history of drug abuse and pregnancy or breastfeeding. Neuropsychological testing was conducted by trained testers in standardised test rooms. Hamilton Depression Rating Scale-6 (HDRS_6_), a subscale of the HDRS_17_ that indexes core MDD symptom, was chosen as the primary clinical outcome with HDRS_17_ as a secondary clinical outcome.

The authors assert that all procedures contributing to this work comply with the ethical standards of the relevant national and institutional committees on human experimentation (protocol: H- 15017713) and with the Helsinki Declaration of 1975, as revised in 2008. The present study is based on baseline data from a longitudinal clinical trial registered at https://www.clinicaltrial.gov (protocol: NCT02869035).

### Hot cognition

#### Affective biases

We used to two tasks from the EMOTICOM test battery to asses biases in emotion processing: the eyes version of the *Emotional* Recognition *Task* (ERT) was used to index biases in basic emotion recognition (i.e. hit rates) as well as misattribution (i.e. false alarm rates) and the *Intensity Morphing* (IM) task was used to assess biases for perceptual detection threshold of emotions in facial expressions (Dam et al., 2019). A modified version of the Verbal Affective Memory Task 24 (Jensen et al., 2016), the *Verbal Affective Memory Task 26* (VAMT-26), was used to assess affective memory biases. Biases were calculated by subtracting negative information scores from positive information scores (e.g. hit rate for recognition of happy faces minus hit rate for recognition of sad faces).

#### Social cognition

We used two tasks from the EMOTICOM test battery to assess social cognition: the *Moral Emotions* (ME) task was used to index moral emotions (guilt and shame) in social situations and the *Social Information Preferenc*e (SIP) task was used to assess preference for social information over non-social information and bias in interpretation of social situations.

### Cold cognition

We used three tasks to assess cold cognition: total word recall in the VAMT-26 was used to assess explicit non-affective verbal memory function; the *Letter* Number *Sequence* (LNS) task was used to assess working memory capacity; and the *Simple Reaction Time* (SRT) task was used to assess reaction time.

Note, a full description of all task including both primary and secondary outcomes can be found in online Supplementary Materials.

### Statistical analysis

#### Group differences

Differences on cognitive performance between depressed patients and healthy controls were assessed with linear regression models with primary cognitive outcome as the dependent variable and age, sex and group coded as a categorical variable as independent variables. The reported *p* values were corrected for 11 tests using the Bonferroni–Holm method. The standardised effect size was estimated for each primary cognitive outcome by computing the Cohen's *d* on the partial residuals relative to the group variable (i.e. after removing the age and sex effects from the cognitive outcome). Normality assumptions were assessed and found to be acceptable for all models except the SRT task outcome. *Post hoc* linear regression analyses were used to investigate secondary outcomes for tasks showing statistically significant group differences. The reported *p* values for the *post hoc* analyses were adjusted to control the family-wise error rate within each task: first the Bonferroni–Holm method was applied (e.g. to eight tests for the ERT secondary outcomes). If the resulting *p* value was smaller than the adjusted *p* value for the primary task outcome, then the adjusted *p* value of the primary task outcome was used for this secondary outcome. This was done to ensure that the adjusted *p* values were coherent between the main and secondary outcomes when using significance threshold below 0.05.

#### Correlation with symptom severity

In MDD patients, Spearman's rank correlation coefficient was used to assess the relationship between depressive symptom severity assessed with HDRS_6_ and HDRS_17_ and performance on primary cognitive outcomes. The reported *p* values were corrected for 11 tests using the Bonferroni–Holm method.

#### Clustering of cognitive profiles

In an exploratory *post hoc* analysis, we used a K-means cluster analysis to delineate potential groups of cognitive profiles within the depressed cohort (Clatworthy, Buick, Hankins, Weinman, & Horne, 2005). The input into the cluster analysis was restricted to the primary cognitive outcomes found to characterise the depressed state, i.e. those outcomes that differed with statistical significance between the depressed patients and healthy controls. The cognitive outcomes were standardised to *z*-scores and the number of clusters and the position used to initialise the K-means algorithm were obtained using a hierarchical clustering algorithm (see online Supplementary Material) (Milligan, 1980). The groups derived from the K-means analysis were compared on clinical and demographic factors using Analysis of Variance (ANOVA) for continuous outcomes and the χ^2^ test for binary outcomes (i.e. sex).

#### Outliers and missing data

Outliers were defined as observations 1.5 Interquartile Range (IQR) above or below the 1^st^ quartile or 3^rd^ quartile respectively. Each outlier was qualitatively evaluated based on notes from the testing session and congruency with scores on outcomes from the same task as well as outcomes from similar tasks. In total, 38 outlying data points were detected (representing 1.8% of all observations across the 11 primary cognitive outcomes); 37 were deemed to be ‘true outliers’ and kept in the analysis while one patient outlier from the LNS task was excluded as the patient had misunderstood the test instructions. Importantly, we found that none of the reported estimates changed critically when all outliers were removed. Note, in the SRT task two patient scores were so extreme (8.1 and 16.1 IQR above the 1^st^ quartile respectively) that independent of their potential neurobiological meaningfulness they were capped to one and two units above the third highest score respectively; this allowed the data to be included without losing their rank or impacting the group estimates unduly. Missing data included IM data from one patient, VAMT-26 data from six patients, SIP data from three controls, LNS data from nine patients and one control and SRT data from one patient and 37 controls.

## Results

### Descriptive

While there was no significant age difference between the two groups, the proportion of females was significantly higher in the patient group (73.9%) compared to the control group (50.4%) (Table 1). This reflects the well-documented higher prevalence of depression in females in the general population; although notably the proportion of females in the present depressed sample was higher than the ~60% reported for European countries in a recent report by the World Health Organization (WHO, 2017). In accordance with the inclusion criteria, all depressed patients had a HDRS_17_ score above 17, indicating moderate to severe depression. IQ was assessed with the Reynolds Intellectual Screening Test (Reynolds, 2011) and all study participants scored within the normal range (see Supplementary Materials). Cognitive performance did not differ between patients with first-episode (*N* = 41) and recurrent depression (*N* = 51) on any task outcome (all *p_corrected_* > 0.75; see online Supplementary Materials for a full overview).

**Table 1.** Descriptive data

	Depressed patients (*n* = 92)	Healthy controls (*n* = 103)	*p* value
Age in years	27.3 ± 8.1 (18–57)	28.7 ± 7.3 (18–48)	0.19
Male/female	25/68	51/52	<0.001
MDI	34.5 ± 7.2 (16–50)^a^	4.9 ± 3.9 (0–20)^b^	<0.001
HDRS_6_	12.4 ± 1.6 (7–17)	–	–
HDRS_17_	22.8 ± 3.4 (18–31)	–	–

Age, sex and self-rated depressive symptoms indexed with the Major Depressive Inventory (MDI) are reported for both depressed patients and healthy controls.For depressed patients, clinically rated depressive symptoms indexed with the Hamilton Depression Rating Scale 6 and 17 (HDRS_6_ and HDRS_17_) are also reported. Values are presented as mean ± sd with range in brackets. Group differences were assessed with an independent *t* test for age; χ^2^ test for sex; and Mann–Whitney *U* test for MDI.

a *N* = 90 due to missing questionnaire data.

b *N* = 102 due to missing questionnaire data.

#### Group differences

*Hot cognition*: In the ERT task, the affective bias expressed by the depressed patients was 11.1 percentage points more negative for recognition rates (*p_corrected_* = 0.03) and 7.8 percentage points more negative for misattribution rates (*p_corrected_* = 0.02) compared to healthy controls. Likewise, the affective bias for emotion detection threshold in the IM task was 7.9 percentage points more negative for the depressed patients (*p_corrected_* < 0.001) while no substantial difference in bias was observed for affective verbal memory in the VAMT-26 (*p_corrected_* = 1.00). When asked to identify with cartoon characters in negative social situations in the ME task, the depressed patients also reported stronger experiences of negative moral emotions equivalent to 0.5 points on a seven-point Likert scale for both guilt (*p_corrected_* < 0.001) and shame (*p_corrected_* < 0.001) compared to healthy controls. We observed no substantial group differences in choice of social information (*p_corrected_* = 1.00) nor bias in interpretation of social situations (*p_corrected_* = 1.00) in the SIP task.

**Fig. 1.**
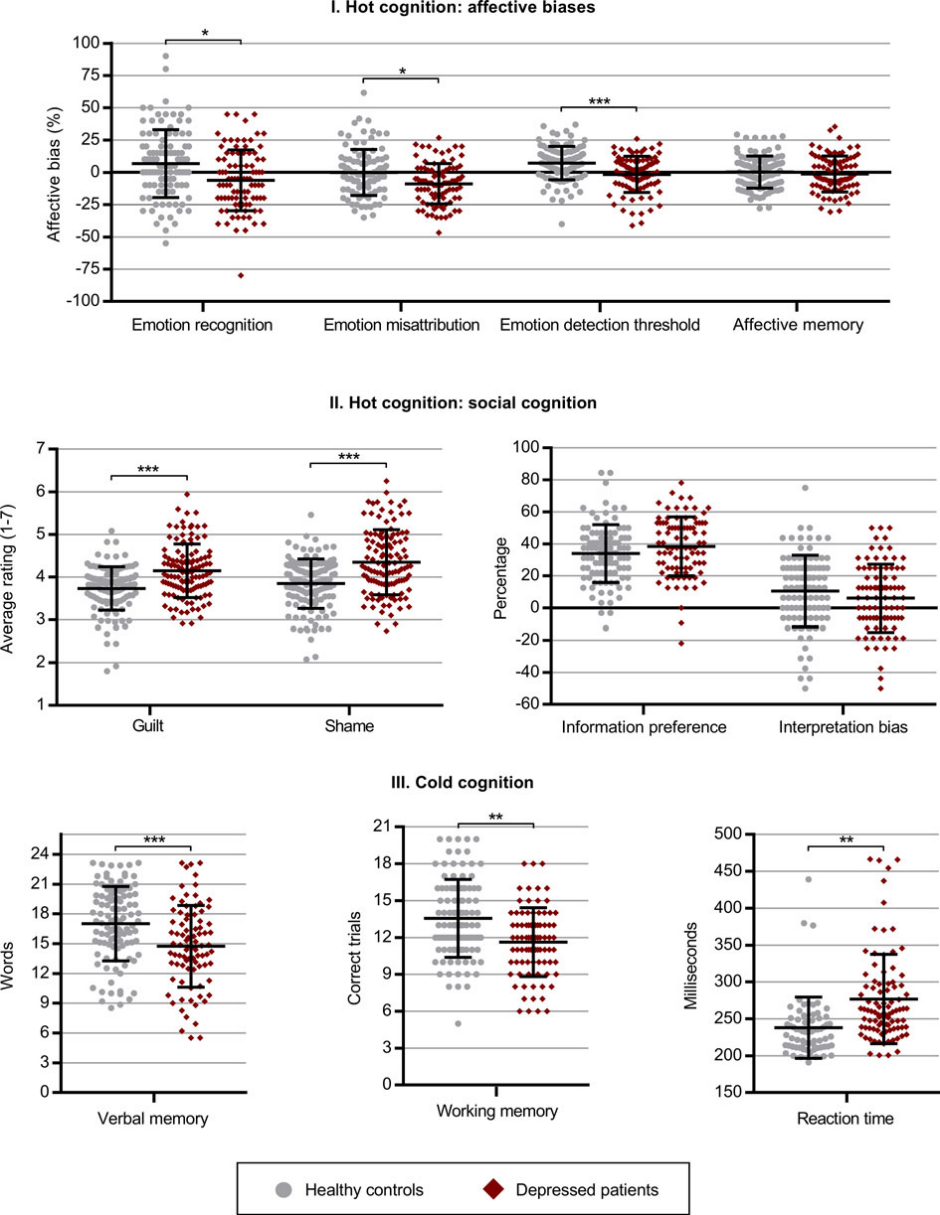
Group differences on affective, social and cold cognitive outcomes between depressed patients and healthy controls. (I) Affective cognition: Recognition = affective bias for hit rate in the Emotional Recognition Task (patients *n* = 92, controls *n* = 103); Misattribution = affective bias for false alarm rate in the Emotional Recognition Task (patients *n* = 92, controls *n* = 103); Detection threshold = affective bias for the Intensity Morphing Task (patients *n* = 91, controls *n* = 103); Affective memory = affective bias for the Verbal Affective Memory Task 26 (patients *n* = 86, controls *n* = 103). (II) Social cognition: Guilt = average ratings of guilt in the Moral Emotions task (patients *n* = 91, controls *n* = 103); Shame = average ratings of shame in the Moral Emotions task (patients *n* = 91, controls *n* = 103); Information preference = choice of theory of mind-related information relative to facts in the Social Information Preference task (patients *n* = 89, controls *n* = 100); Interpretation bias = affective bias in choice of outcome in the Social Information Preference task (patients *n* = 89, controls *n* = 100). (III) Cold cognition: Verbal memory = Total recall score for the Verbal Affective Memory Task (patients *n* = 85, controls *n* = 103); Working memory = Letter-Number Sequence task (patients *n* = 83, controls *n* = 103); Reaction time = Simple Reaction Time (patients *n* = 91, controls *n* = 66). All models were corrected for age and sex. *p < 0.05, **p < 0.01, ***p < 0.001.

*Cold cognition*: The depressed patients recalled a total of 2.6 fewer words in the VAMT-26 (*p_corrected_* < 0.001) independent on affective valence; successfully sorted 1.7 fewer sequences on the working memory task (LNS, *p_corrected_* *=* 0.002); and exhibited 30.7 ms slower reaction time (SRT, *p_corrected_* = 0.006) compared to the healthy controls. Note, as model assumptions for normality were not met for the SRT outcome, we used bootstrapping to determine the reported *p* value. In addition, we also conducted a quantile regression analysis to assess whether the reported results were robust to outliers and found a similar effect (estimated group effect = 20.7 ms, *p* value = 0.003) (Fig. 1).

**Fig. 2.**
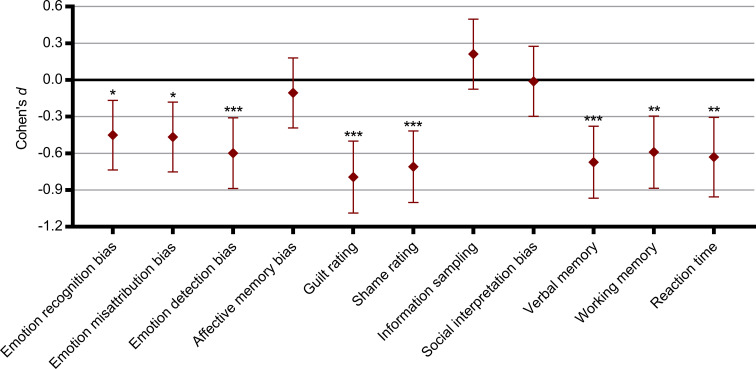
Summary of differences in performance across cognitive domains for depressed patients relative to healthy controls. Zero represents the healthy control group and differences are expressed as Cohen's *d* effect sizes. Error bars denote 95% confidence intervals (95% CI). Recognition bias = affective bias for hit rate in the Emotional Recognition Task; Misattribution bias = affective bias for false alarm rate in the Emotional Recognition Task; Detection bias = affective bias for the Intensity Morphing task; Affective memory bias = affective bias for the Verbal Affective Memory Task 26; Guilt rating = average guilt rating from the Moral Emotions task; Shame rating = average shame rating from the Moral Emotions task; Information sampling = choice of theory of mind-related information relative to facts in the Social Information Preference task; Interpretation bias = affective bias in choice of outcome in the Social Information Preference task; Verbal memory = total recall from the Verbal Affective Memory Task 26; Working memory = Letter-Number Sequence task; Reaction time = Simple Reaction Time task. * *p* < 0.05, ** *p* < 0.01, *** *p* < 0.001.

Compared to healthy controls, MDD patients were more likely to incorrectly identify other emotions as sadness in the ERT task and continued to perceive sadness at lower intensity levels (decrease condition) in the IM task. In the ME task, patients also reported higher levels of guilt and shame in social scenarios where they identified with characters who accidentally harmed another person and increased guilt and scenarios where they identified with the victim of either accidental or intentional harm. Lastly, patients exhibited deficits for both immediate, short-term and long-term non-affective verbal memory compared to controls in the VAMT-26 (Fig. 2).

#### Correlation with depression severity

Correlations between cognitive performance and clinically rated depressive symptoms within the depressed group ranged from weak to negligible on all tasks and were statistically non-significant [HDRS_6_, *ρ* (−0.2; 0.2), all *p_corrected_* > 0.42; HDRS_17_, *ρ* (−0.2; 0.2), all *p_corrected_* > 0.44] (see online Supplementary Materials for a full overview).

#### Clustering of cognitive profiles

**Fig. 3.**
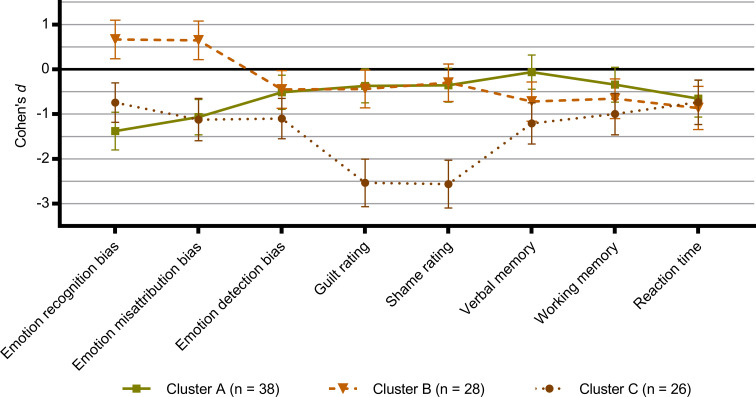
Clusters of cognitive profiles within the cohort of depressed patients (*N* = 92) based on the eight cognitive outcomes that showed a significant group difference between depressed patients and healthy controls. Zero represents the healthy control group and differences are expressed as Cohen's *d* effect sizes. Error bars denote 95% confidence intervals (95% CI). Recognition bias = affective bias for hit rate in the Emotional Recognition Task; False alarm bias = affective bias for false alarm rate in the Emotional Recognition Task; Detection bias = affective bias for the Intensity Morphing task; Affective memory bias = affective bias for the Verbal Affective Memory Task 26; Guilt rating = average guilt rating from the Moral Emotions task; Shame rating = average shame rating from the Moral Emotions task; Information sampling = choice of theory of mind-related information relative to facts in the Social Information Preference task; Interpretation bias = affective bias in choice of outcome in the Social Information Preference task; Verbal memory = total recall from the Verbal Affective Memory Task 26; Working memory = Letter-Number Sequence task; Reaction time = Simple Reaction Time task.

Based on the eight cognitive outcomes which showed a significant group difference, an initial hierarchical cluster analysis was run that indicated a three-cluster solution for cognitive profiles within the depressed group. The clustering centroids from the hierarchical cluster analysis were subsequently used to initialise a K-means analysis that converged within six iterations (Fig. 3).

There were no statistically significant differences between the clusters on age (*p* = 0.58) or sex (*p* = 0.71). The three clusters differed significantly on severity of core depressive symptoms indexed with HDRS_6_ [*F*(2, 90) = 4.1, *p* = 0.02] with patients from Cluster C (13.1 ± 1.9, mean ± s.d.) having higher scores than Cluster A (12.2 ± 1.6, mean ± s.d.) and B (11.9 ± 1.3, mean ± s.d.). The same pattern was present in severity of broad depressive symptoms indexed with HDRS_17_ (Cluster A, HDRS_17_ scores = 22.1 ± 3.2, mean ± s.d.; Cluster B, HDRS_17_ scores = 23.0 ± 3.1, mean ± s.d.; Cluster C, HDRS_17_ scores = 23.6 ± 3.8, mean ± s.d.), but did not reach statistical significance (*p* = 0.19).

## Discussion

We here map the presence and magnitude of both hot and cold cognitive disturbances in a large cohort of antidepressant-free patients with a moderate to severe depressive episode. We found small to moderately sized negative biases in emotion processing but not in explicit verbal memory, large increases in experience of negative social emotions but no detectable differences in preference between social and non-social information or interpretation of ambiguous social situations. We also observed moderate impairment of cold cognitive functions including working and verbal memory and moderate slowing of reaction time. We found no direct link between depressive symptom severity and patient performance on any of the single task domains. Using an exploratory and data-driven approach, we identified three clusters with distinct cognitive profiles within the cohort of depressed patients: Cluster A was characterised by disturbances in hot but not cold cognition; Cluster B was characterised by positive biases and moderate deficits in cold cognitive domains; and Cluster C was characterised by large deficits across both hot and cold cognitive domains including extreme scores of guilt and shame.

### Affective biases

As expected, the depressed patients exhibited negative affective biases across all emotion processing outcomes including recognition, misattribution and perceptual detection threshold. While abnormal processing of facial expressions is well-established in the MDD literature, the underlying cognitive mechanisms are still unclear (Elliott et al., 2011). Indeed, the findings from our study emphasise that the negative biases exhibited by the depressed patients must be understood *relative* to healthy controls. For example, while the depressed patients exhibited a clear negative affective bias in emotion misattribution, the negative bias observed for emotion detection threshold predominantly reflected the loss of positive bias exhibited by the healthy controls (see Fig. 1). Another line of research from attention paradigms suggests that affective bias in emotion processing is related to reduced orientation towards positive stimuli combined with an inability to disengage from negative stimuli (Armstrong & Olatunji, 2012). This aligns with our findings from the IM task as patients continued to perceive sadness at much lower intensity levels in the decrease condition compared to healthy controls.

Notably, we did not observe any negative affective bias in verbal memory performance. The concept of a mood-congruent memory bias was first proposed by Bower (1981) and posits that individuals will remember information that matches their current emotional state better than information that is not mood-congruent. Although this theory is relatively well supported by empirical studies investigating autobiographical (Köhler et al., 2015) and implicit (Gaddy & Ingram, 2014) types of memory, evidence for a bias in explicit, non-self-referential memory remains inconclusive. In fact, only a handful of studies have specifically investigated this type of memory (typically using valanced word-lists) in depression with some reporting a negative bias (Bradley, Mogg, & Williams, 1995; Neshat-Doost, Taghavi, Moradi, Yule, & Dalgleish, 1998; Watkins, Mathews, Williamson, & Fuller, 1992) while others suggest a positive bias (Calev, 1996; Danion, Kauffmann-Muller, Grange, Zimmermann, & Greth, 1995; Zupan, Žeželj, & Andjelković, 2017). In contrast to word memory, there have been attentional biases to negative words reported for unmedicated depressed subject (Beavers et al., 2013). The majority of memory studies had very small sample sizes and used different cognitive tasks and different depression criteria, which may contribute to the lack of consensus. We here present data from one of the largest study populations to date of well-characterised depressed patients which suggest that MDD symptomatology is not related to mood-congruent memory bias in explicit non-self-referential affective memory. Future studies should therefore consider using cognitive tasks assessing autobiographical and implicit memory as they may be more sensitive to affective memory disturbances in MDD.

### Social cognition

Feelings of excessive shame and guilt are common in MDD and critically contribute to low self-esteem and social withdrawal (Mills et al., 2015). In the most severe cases they can even reach the threshold of psychosis (Lake, 2008). In particular contextual-maladaptive guilt (i.e. exaggerated guilt related to uncontrollable events) and generalised guilt (i.e. guilt divorced from concrete contexts) as well as external shame (i.e. shame based on beliefs about other people's opinions) are strongly associated with depressive symptoms (Kim, Thibodeau, & Jorgensen, 2011). Notably, in the ME task we observed most group differences between the depressed patients and the healthy controls in moral scenarios where the participants were asked to identify with the victim of harm (see Table 2). In fact, the level of shame and guilt reported by the patients appeared most pronounced when they were the victim of intentional rather than accidental harm, likely reflecting a disengaging of other-blaming schemata in favour of maladaptive self-blaming and internalising schemata. Clearly, this represents a critical target to address in psychotherapy, e.g. cognitive behavioural therapy (CBT).

**Table 2.** Group differences between depressed patients and healthy controls on secondary cognitive outcomes

	Depressed patients		Healthy controls		*Β*	*p* value	*p_corrected_*
	Mean ± sd	Range	Mean ± sd	Range			
Emotion recognition task							
Recognition – Happy	72.4 ± 17.8	15.0–100.0	78.0 ± 16.7	20.0–100.0	−5.0	5.25 × 10^−2^	0.21
Recognition – Sad	78.6 ± 16.6	30.0–100.0	71.3 ± 18.9	10.0–100.0	6.2	2.01 × 10^−2^	0.14
Recognition – Angry	62.5 ± 12.8	30.0–95.0	66.0 ± 11.4	40.0–90.0	−3.7	4.15 × 10^−2^	0.21
Recognition – Fearful	73.5 ± 11.7	35.0–95.0	75.4 ± 14.8	5.0–100.0	−2.4	2.18 × 10^−1^	0.44
Misattribution – Happy	10.9 ± 8.4	0.0–36.7	15.0 ± 12.0	0.0–63.3	−3.5	2.37 × 10^−2^	0.14
Misattribution – Sad	19.8 ± 9.3	0.0–48.3	15.1 ± 8.6	0.0–41.7	4.3	1.52 × 10^−3^	0.03
Misattribution – Angry	3.0 ± 3.7	0.0–18.3	3.7 ± 5.8	0.0–50.0	−0.5	4.58 × 10^−1^	0.46
Misattribution – Fearful	3.9 ± 6.0	0.0–28.3	2.6 ± 3.8	0.0–26.7	1.4	5.13 × 10^−2^	0.21
Intensity morphing task^a^							
Increase – Happy	51.1 ± 16.1	23.2–92.9	46.9 ± 14.9	12.5–88.1	4.5	5.09 × 10^−2^	0.31
Increase – Sad	53.9 ± 14.0	23.2–82.1	60.2 ± 15.2	17.9–89.3	−5.0	2.08 × 10^−2^	0.15
Increase – Angry	52.8 ± 13.2	21.4–85.7	55.6 ± 15.6	17.9–92.9	−1.9	3.68 × 10^−1^	0.82
Increase – Fearful	56.1 ± 16.2	21.4–100.0	61.0 ± 16.7	21.4–100.0	−2.9	2.31 × 10^−1^	0.82
Increase – Disgusted	55.5 ± 15.3	19.6–92.9	57.2 ± 14.7	17.9–89.3	0.06	9.80 × 10^−1^	0.98
Decrease – Happy	29.0 ± 13.2	2.4–66.1	30.9 ± 16.2	0.0–75.0	−2.8	2.06 × 10^−1^	0.82
Decrease – Sad	22.9 ± 12.2	0.0–69.6	31.9 ± 12.3	5.4–66.1	−9.2	1.00 × 10^−6^	<0.001
Decrease – Angry	21.9 ± 13.5	0.0–100.0	25.5 ± 12.2	3.6–62.5	−3.5	7.29 × 10^−2^	0.36
Decrease – Fearful	24.4 ± 13.6	0.0–92.9	29.7 ± 11.4	0.0–66.1	−5.0	7.37 × 10^−3^	0.06
Decrease – Disgusted	17.2 ± 9.9	0.0–44.6	22.0 ± 12.4	0.0–67.9	−4.7	6.29 × 10^−3^	0.06
Moral emotions task							
Agent/intentional – Guilt	6.3 ± 0.6	4.2–7.0	6.1 ± 0.8	2.5–7.0	0.1	2.00 × 10^−1^	0.40
Agent/accident – Guilt	6.1 ± 0.8	3.6–7.0	5.6 ± 0.8	2.0–7.0	0.4	4.53 × 10^−4^	0.002
Victim/intentional – Guilt	2.5 ± 1.1	1.0–5.3	1.7 ± 0.7	1.0–3.8	0.8	1.67 × 10^−8^	<0.001
Victim/accident – Guilt	2.0 ± 0.9	1.0–5.0	1.5 ± 0.6	1.0–4.4	0.5	8.00 × 10^−6^	<0.001
Agent/intentional – Shame	6.1 ± 0.8	4.3–7.0	6.0 ± 0.9	2.5–7.0	0.03	8.00 × 10^−1^	0.80
Agent/accident – Shame	5.9 ± 0.8	3.6–7.0	5.5 ± 0.9	2.0–7.0	0.3	7.68 × 10^−3^	0.02
Victim/intentional – Shame	3.5 ± 1.3	1.0–6.5	2.5 ± 1.0	1.0–4.8	0.9	2.48 × 10^−7^	<0.001
Victim/accident – Shame	2.1 ± 1.1	1.0–6.1	1.5 ± 0.6	1.0–4.0	0.6	7.72 × 10^−7^	<0.001
Verbal affective memory task 26^b^							
Immediate recall	14.6 ± 3.3	6.6–23.4	16.0 ± 3.0	8.2–21.0	−1.6	7.17 × 10^−4^	0.001
Short-term recall	14.7 ± 4.6	4.0–24.0	17.2 ± 4.5	7.0–25.0	−2.8	3.40 × 10^−5^	<0.001
Delayed recall	15.0 ± 4.9	4.0–26.0	17.9 ± 4.2	9.0–26.0	−3.2	3.00 × 10^−6^	<0.001

Raw *p* values as well as corrected *p* values are reported (see Method sections for description). *β*-Values represent difference in scores between patients and healthy controls once age and sex has been accounted for.

a Depressed patients *n* = 91.

b Depressed patients *n* = 86.

Recent evidence suggests that ToM, i.e. the ability to attribute mental states to other people, is impaired in MDD and linked to depressive symptom severity (Bora & Berk, 2015). While we did not have a direct measure ToM in the present study, the SIP task indexes the preference for choosing social information (thoughts or facial expression) over non-social information (facts) when interpreting socially ambiguous situations. We did not observe any differences between patients and healthy controls, suggesting that it is not a lack of attention towards or a preference away from social information that is causing the reported ToM deficits in MDD. This aligns with reports that dysphoria is associated with slightly *increased* sensitivity to social cues required for ToM (Harkness, Sabbagh, Jacobson, Chowdrey, & Chen, 2005). Our depressed patients did not exhibit negative bias in the interpretation of social situations. We speculate that this may partially be related to the task design; in several scenarios the negative interpretation had paranoid components (e.g. believing a colleague is poisoning a cup of tea) and might be too extreme to capture the more subtle negative biases in MDD.

### Cold cognition

We were able to replicate previously reported impairments in verbal memory, working memory and reaction time (Bennabi et al., 2013; Goodall et al., 2018; Lee et al., 2012; Marazziti, Consoli, Picchetti, Carlini, & Faravelli, 2010; Rock et al., 2014) showing moderate effect sizes. The difference in number of remembered words in the VAMT-26 between patients and healthy controls also appeared to become progressively larger across the three time points (immediate recall, 1.6 words; short-term recall 2.8 words, and long-term recall, 3.2 words). This could potentially indicate that initial learning is less affected than long-term memory or alternatively reflect effects of fatigue and/or apathy in the depressed patients (Marazziti et al., 2010).

### Correlation with depression severity

We were unable to identify a clear association between any of the individual cognitive task domains and depression severity indexed with HDRS_6_ or HDRS_17_ scores. This suggests that cognitive disturbances may not simply be an extension of the ‘classic’ core mood and somatic symptoms in MDD but rather represent distinct characteristics of the depressive pathology. Furthermore, while cognitive disturbances may be largely independent from symptom severity during the depressive episode, they are still associated with long-term clinical and functional outcomes (Cicchetti, 1994; Collins et al., 2011) and thus hold promise as a relevant stratification tool for the identification of clinically meaningful subgroups in MDD. Indeed, empirical trials are currently investigating the benefits of cognition-based tools for optimising treatment in MDD (Kingslake et al., 2017). However, more work is still needed to evaluate the clinical value of such approaches.

### Clustering of cognitive profiles

In an exploratory analysis, we identified three clusters of cognitive profiles in the depressed patients. Cluster A was the largest group (*n* = 38) and characterised patients with strong negative biases in emotion recognition and misattribution but no substantial deficits in cold cognitive domains apart from slowed reaction time. Cluster B (*n* = 28) conversely characterised patients with positive biases in emotion processing and moderate deficits across all cold cognition domains. Lastly, Cluster C (*n* = 26) characterised patients who had large deficits across both hot and cold cognitive domains and in particular extremely high ratings of shame and guilt. These findings not only suggest a dissociation between the presence of hot and cold cognitive deficits in MDD, as illustrated by the differences between Cluster A and B, but also the existence of a subgroup of patients with severe global cognitive deficits represented by Cluster C.

To our knowledge, only two other studies have used cluster analysis to identify cognitive profiles in MDD: the large iSPOT-D trial (*N* = 1008) (Etkin et al., 2015) and a smaller study (*N* = 50) in patients with first-episode depression (Vicent-Gil et al., 2018). Both studies identified two clusters based on performance on cold cognitive tasks: a large cluster of cognitive intact patients and a smaller cluster of cognitive impaired patients. Notably, the proportion of cognitive impaired patients reported in both studies was 25–26%, closely matching the size of the globally impaired Cluster C in the present cohort (~28%). This further aligns with previous reports that only a small proportion of patients experience pronounced impairments in cognitive performance with estimates ranging between 21 and 44% depending on the cognitive measures and cut-off criteria used (Gualtieri & Morgan, 2008; Iverson, Brooks, Langenecker, & Young, 2011; McIntyre et al., 2017). Importantly, none the above studies included measures of affective biases or social cognition and may therefore have overlooked the presence of Cluster A type patients who exhibit strong negative biases in emotion processing but little to no deficits in cold cognitive domains. This highlights the importance of characterising *both* cold and hot cognitive disturbances in MDD concurrently.

Interestingly, the degree of cognitive disturbances across the three clusters partly mirrored the severity of depressive symptoms within the clusters, i.e. Cluster C had overall higher levels of depressive symptoms compared to Cluster A and B. This indicates that these cognitive profiles are able to capture MDD characteristics not captured by any individual task domain. Future studies should evaluate whether such cluster labelling, in addition to single cognitive domain information, may be useful for guiding antidepressants treatment choices and/or identify patients who will benefit from augmentation with e.g. cognitive remediation (Maruff & Jaeger, 2016) or cognitive enhancers (Bowie, Gupta, & Holshausen, 2013).

### Methodological considerations

Some methodological limitations should be considered: (1) The depressed patients and healthy controls were unevenly matched on sex and because of the recruitment and inclusion procedures, the healthy controls likely represent very high-functioning individuals which may have inflated the observed differences on cognitive outcomes between the two groups. (2) We did not correct for the effect of IQ or education in the analyses as previous reports indicate that IQ measurement (Goss, Kaser, Costafreda, Sahakian, & Fu, 2013; Miskowiak et al., 2014) and education dropout rates (Marazziti et al., 2010) are affected by depressive symptoms. None of the reported estimates changed critically when IQ or education were included in the models (for corrected estimates see online Supplementary Materials). (3) Because the wordlist in the VAMT-26 contained both positively and negatively valanced words, the total word recall score does not represent a ‘pure’ cold measure of explicit memory. (4) Due to the limited stamina of the MDD patients, we had to restrict the number of cognitive domains tested; as a consequence, we did not collect data on e.g. attention or higher-executive functions despite their relevance in MDD pathology.

## Conclusion

The current study represents one of the most comprehensive investigations into hot and cold cognitive impairment in a large, well-characterised and antidepressant-free cohort of depressed patients to date. This allowed us to assess and directly compare the magnitude and patterns of impairment across a broad range of cognitive domains as well as investigate the presence of clusters of distinct cognitive profiles in depression. It is also the first time tasks from the EMOTICOM test battery have been applied and shown to be sensitive to MDD pathology in a patient cohort. Overall, our findings highlight the importance of including both hot and cold cognitive domains in investigations into MDD and further suggest that cognitive measures capture features beyond those reflected by depression severity. While cognitive disturbances are not present in all patients, they do represent significant impairments in identifiable and large subgroups of patients that may benefit from augmentation with cognition targeted treatments. Thus, we argue that cognition-based tools hold promise as clinically useful stratification aids in the care of depressed patients.

## Supplementary material

For supplementary material accompanying this paper visit https://doi.org/10.1017/S0033291720000938. click here to view supplementary material
