# Supplementary material for: Hot and cold cognitive disturbances in antidepressant-free patients with major depressive disorder: a NeuroPharm study
Source: Psychol Med. 2020 Apr 22;51(14):2347–56. doi: 10.1017/S0033291720000938 (PMC8506354; doi:10.1017/S0033291720000938)
Supplement: Supplementary file 1 [file S0033291720000938sup001.docx]

**Supplementary Materials**

1. Description of cognitive tasks and outcomes………………………………………………………. p. 2

2. Cluster analysis…………………………………………………………………………………….. p. 6

3. First-episode vs recurrent depression……………………………………….……………………… p. 9

4. Correlation between cognitive scores and clinical symptoms……………………………………. p. 11

5. Cognitive task scores across groups………………………………………………………………. p. 12

6. Effect of IQ and education on group difference estimates……………………………………….. p. 14

**1. Description of cognitive tasks and outcomes**

**Hot cognition**

**Emotion recognition**

The eyes version of the Emotional Recognition Task (ERT) from the EMOTICOM test battery was used to assess recognition of facial expressions ([Bland et al., 2016](#_ENREF_1); [Dam et al., 2019](#_ENREF_2)). Participants were asked to determine which emotion (happy, sad, angry or fearful) was being expressed by a pair of eyes shown briefly (250ms) on a computer screen.


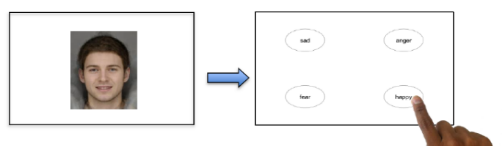

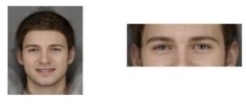


Main outcomes: Affective bias for recognition (hit rate i.e. percentage of trials in which a given emotion was correctly identified) and misattribution (false alarm rate i.e. percentage of trials in which a given emotion was wrongly identified). Affective bias was calculated as: *Hit_happy_ - Hit_sad_* and *FalseAlarm_happy_ - FalseAlarm_sad_*

Secondary outcomes: Hit rate and false alarm rate for each emotion: happy, sad, angry and fearful.

**Emotion detection threshold**


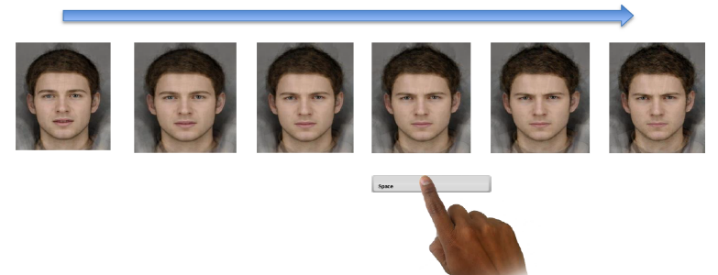
The Intensity Morphing task (IM) from the EMOTICOM test battery was used to assess the perceptual threshold for detection of emotions in facial expressions ([Bland et al., 2016](#_ENREF_1)). Participants were asked to indicate when they were either able to detect (increase condition) or no longer detect (decrease condition) a given emotion (happy, sad, angry, fearful, or disgusted) on a face with a slowly morphing expression.

Main outcomes: Affective bias in percentage averaged across both increase and decrease condition calculated as: *DetectionThreshold_sad_ - DetectionThreshold_happy_*. To ensure that a positive score indicate a positive bias, ‘Happy’ was subtracted from ‘Sad’ for the IM task because low scores indicate a low detection threshold, i.e., higher perceptual sensitivity to the presence of the emotion.

Secondary outcomes: Detection threshold for each of the five emotions for both the increase and decrease condition.

**Affective verbal memory**


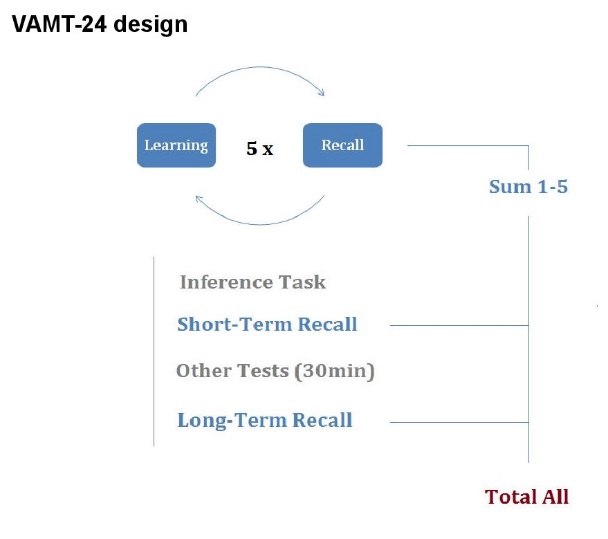
A modified version of the Verbal Affective Memory Task 24 with 26 words (Verbal Affective Memory Task 26; VAMT-26) was used to assess learning and memory of affective words ([Jensen et al., 2016](#_ENREF_3)). A list of words (10 positive, 10 negative and 6 neutral) were presented briefly one by one in a pseudo-randomized order on a computer screen. After viewing the list, the participants were asked to verbally recall as many words as possible in no specific order. Immediate recall was determined as average number of words remembered across five viewings of the list, short-term recall as number of words remembered after viewing an interference list, and delayed recall as number of words remembered after a span of 30 minutes.

Main outcome: Affective bias for total word recall (i.e. average words remembered across immediate, short-term, and delayed recall) calculated as: *WordScore_positive_ – WordScore_negative_*

**Moral emotions**


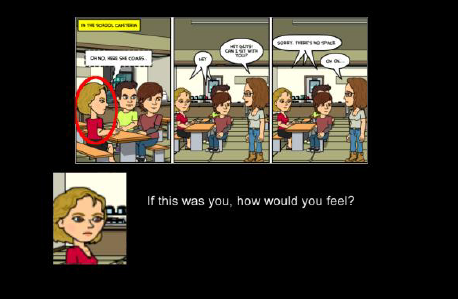
The Moral Emotions Task (ME) was used to assess moral emotions in social situations. Participants were shown cartoons in which one person either intentionally or accidentally causes another person harm. Participants were instructed to imagine themselves as either the agent (i.e. the person causing the harm) or the victim and rate how guilty and ashamed they would feel.

Main outcomes: Average rating across all conditions for guilt and shame.

Secondary outcomes: Ratings of guilt and shame for each of the four conditions: Agent intentionally causing harm; agent accidentally causing harm; victim of intentional harm; and victim of accidental harm.

*Social information preference*

The Social Information Preference task (SIP) was used to assess information sampling and interpretation of social situations. Participants were shown cartoons depicting social interactions in which several pieces of information were hidden (thoughts, facial expression, and facts/items). Participants were instructed to pick four pieces of information to help them interpret the situation and to choose between a positive, neutral, and negative outcome.

Main outcomes: Preference for social information over non-social information calculated as: *Choicet_houghts_ + Choice_faces_ – Choice_facts_* and affective bias in choice of scenario outcome calculated as: *Outcome_positive_ – Outcome_negative_*

**Cold cognition**

**Explicit verbal memory**

VAMT-26 was used to assess overall verbal memory function.

Main outcome: Total word recall calculated as average number of words (positive, negative and neutral) recalled across immediate, short-term and long-term recall.

**Working memory**


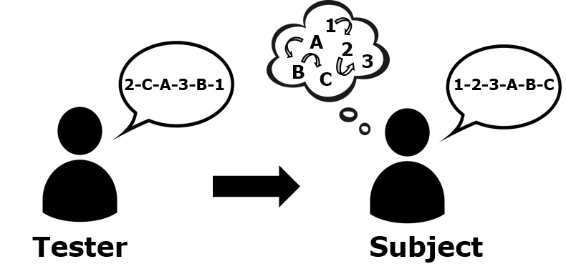
The Letter Number Sequence task (LNS) was used to assess working memory capacity. Participants were asked to remember and mentally sort a sequence of jumbled letters and numbers of increasing length.

Main outcome: Total number of correctly recited sequences with scores ranging from 0-21.

**Reaction time**


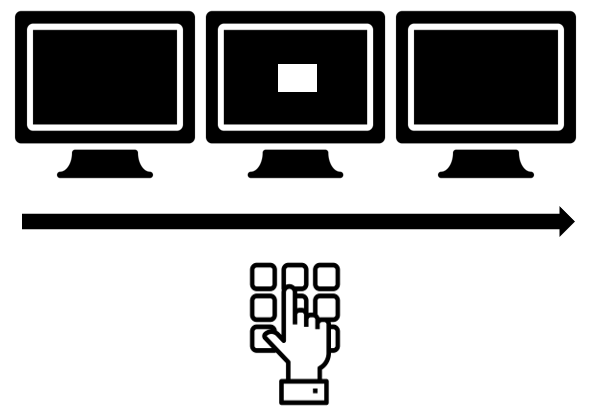
The Simple Reaction Time task (SRT) was used to assess reaction time. Participants were instructed to press a button as fast as possible at the appearance of a white square on a screen. The location of the square did not change but the interval between appearances varied.

Main outcome(s): Reaction time latency in milliseconds.

**References**

Bland, A. R., Roiser, J. P., Mehta, M. A., Schei, T., Boland, H., Campbell-Meiklejohn, D. K., . . . Elliott, R. (2016). EMOTICOM: A Neuropsychological Test Battery to Evaluate Emotion, Motivation, Impulsivity, and Social Cognition. *Front Behav Neurosci, 10*, 25. doi: 10.3389/fnbeh.2016.00025

Dam, V. H., Thystrup, C. K., Jensen, P. S., Bland, A. R., Mortensen, E. L., Elliott, R., . . . Stenbæk, D. S. (2019). Psychometric Properties and Validation of the EMOTICOM Test Battery in a Healthy Danish Population. *Frontiers in Psychology, 10*(2660). doi: 10.3389/fpsyg.2019.02660

Jensen, C. G., Hjordt, L. V., Stenbaek, D. S., Andersen, E., Back, S. K., Lansner, J., . . . Hasselbalch, S. G. (2016). Development and psychometric validation of the verbal affective memory test. *Memory, 24*(9), 1208-1223. doi: 10.1080/09658211.2015.1087573

**2. Cluster analyses**

To ascertain the optimal number of clusters for the K-means cluster analysis, we conducted a Hierarchical Cluster Analysis (HCA) using Ward’s method with squared Euclidian distance as the similarity measure (Ward, 1963). Input into the HCA was *z*-transformed scores from the eight primary cognitive outcomes which was found to significantly differ between depressed patients and healthy controls (i.e., recognition and misattribution rates from the *Emotional Recognition Task*-eyes version, detection threshold from the *Intensity Morphing* task, guilt and shame ratings from the *Moral Emotions* task, verbal memory from the *Verbal Affective Memory Task-26*, working memory from the *Letter Number Sequence* task, and reaction time from the *Simple Reaction Time* task). From the HCA a dissimilarity measure coefficient was obtained (Table S2.1). The change in dissimilarity was displayed against the number of clusters and the optimal number of clusters was determined as the cluster just prior to the largest jump in change in dissimilarity measure coefficient (Figure S2.1). Thus, a three-cluster solution was indicated supported by the produced dendrogram (Figure S2.2). Lastly, the clustering centroids from the three clusters (i.e. group means for each cognitive outcome) were used to initialize the K-means analysis (Milligan, 1980).

**Table S2.1. Agglomeration schedule**

| Number of clusters | Dissimilarity measure  coefficient | Change in dissimilarity measure coefficient | Jump in change of dissimilarity measure coefficient |
| --- | --- | --- | --- |
| 1 | 643.9 | 96.3 | 18.5 |
| 2 | 547.7 | 77.8 | 31.2 |
| 3 | 469.8 | 46.6 | 3.9 |
| 4 | 423.2 | 42.7 | 7.3 |
| 5 | 380.5 | 35.4 | 5.8 |
| 6 | 345.1 | 29.6 | 3.7 |
| 7 | 315.5 | 26.0 | 8.3 |
| 8 | 289.5 | 17.6 | 3.0 |
| 9 | 271.9 | 14.7 | 1.9 |
| 10 | 257.2 | 12.8 | 0.7 |
| 11 | 244.5 | 12.0 | 0.4 |
| 12 | 232.5 | 11.6 | 0.8 |
| 13 | 220.9 | 10.8 | 1.2 |
| 14 | 210.1 | 9.7 | 0.2 |
| 15 | 200.4 | 9.4 | 1.0 |

**Table S2.1.** The table shows the dissimilarity measure coefficients from the agglomeration schedule. In addition, the calculated change in dissimilarity measure coefficient as well as the jump in change of dissimilarity measure coefficient is shown. Note we here only show the coefficients for cluster solutions 1-15 as a larger number of clusters would not be meaningful in the present study.

**Figure S2.1. Changes in dissimilarity measure coefficients**


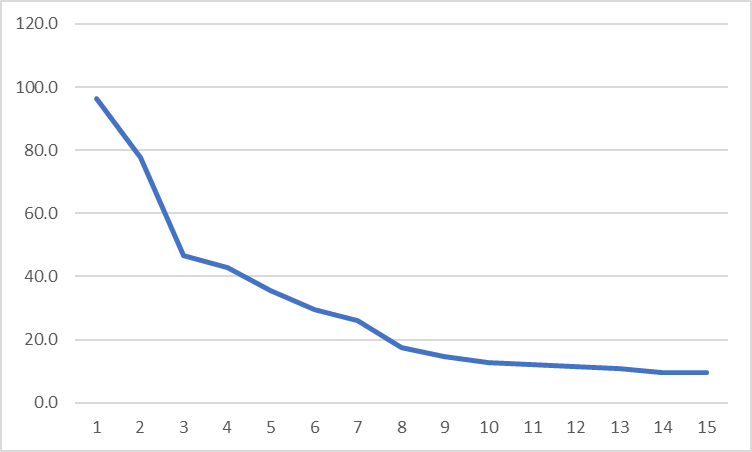


**Figure S2.1.** The graph shows changes in dissimilarity measure coefficient (y-axis) across different numbers of clusters (x-axis). The changes were calculated as the difference in dissimilarity measure coefficient for each added cluster. The red circle indicates the largest and most inconsistent change, suggesting that the clustering process should be stopped at a three-cluster solution.

**Figure S2.2. Dendrogram**


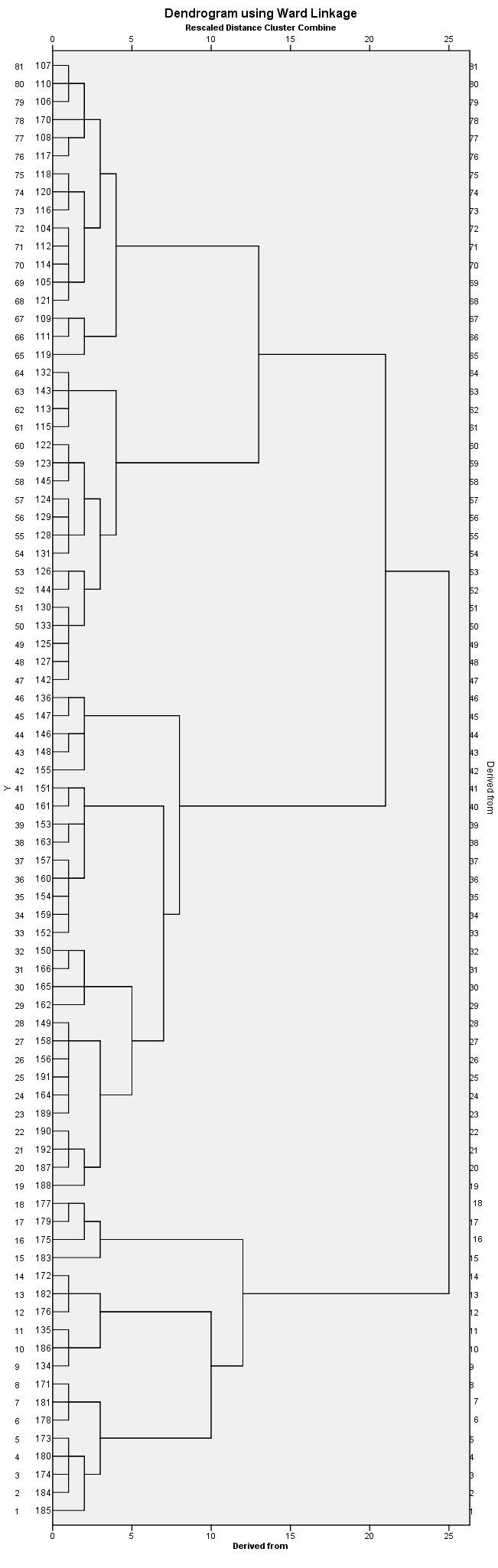


**Figure S2.2.** Dendrogram produced from Hierarchical Cluster analysis based on the scores of depressed patients on eight primary cognitive outcomes. The colored boxes indicate the three clusters suggested by the dendrogram.

**References**

**Milligan, G. W.** (1980). An examination of the effect of six types of error perturbation on fifteen clustering algorithms. *Psychometrica* **45**, 325-342.

**Ward, J. H.** (1963). Hierarchical Grouping to Optimize an Objective Function. *Journal of the American Statistical Association* **58**, 236-244.

**3. First-episode vs recurrent depression**

History of previous depressive episodes was collected based on patient testimony supplemented with information from medical records when possible. Figure S3.1. provides an overview of the number of patients with first-episode depression (N = 41) and recurrent depression (N = 51), including number of previous depressive episodes in the latter group.

**Figure S3.1.** Distribution of MDD patients (N = 92) with first episode depression and recurrent depression (i.e. ≥ 2 episodes). Note for = 10 patients, the exact number of depressive episodes could not be verified beyond recurrent depression (i.e. more than one episode).

Table S3.1. shows descriptive characteristics of first-episode and recurrent depression patients.

| **Table S3.1 History of depressive episodes** | | | | |  |  |  |
| --- | --- | --- | --- | --- | --- | --- | --- |
|  | First-episode (N = 41) | |  | Recurrent (N = 51) | |  | *p* |
|  | Mean ± SD | Range |  | Mean ± SD | Range |  |  |
| Age | 28.8 ± 10.6 | 18–57 |  | 26.0 ± 5.3 | 18–43 |  | 0.73 |
| Male/female | 13/28 | |  | 12/39 | |  | 0.40 |
| HDRS6 | 12.4 ± 1.7 | 8–17 |  | 12.3 ± 1.6 | 7–17 |  | 0.89 |
| HDRS_17_ | 22.7 ± 3.2 | 18–31 |  | 22.9 ± 3.5 | 18–31 |  | 0.82 |
| **Table S3.1.** Age, sex distribution and depressive symptoms severity (Hamilton Depressive Rating Scale 6, HDRS_6_) between patients with first-episode depression and patients with recurrent depression. Group differences were assessed using Mann Whitney U-tests and χ^2^ test (for sex). | | | | | | | |

There here was no statistically significant difference between the number of patients with first-episode vs recurrent depression across the three cognitive profile clusters, *χ^2^*(2, *N* = 92) = 1.4, *p* = 0.5.

| **Table S3.2. Depressive history and cognitive performance** | | | | | | | |  |
| --- | --- | --- | --- | --- | --- | --- | --- | --- |
|  | First-episode (N = 41) | |  | Recurrent (N = 51) | | *β* | *p* | *p_corrected_* |
|  | Mean ± SD | Range |  | Mean ± SD | Range |  |  |  |
| *Hot cognition I: affective biases* | | | | |  |  |  |  |
| Emotion recognition | -9.5 ± 24.2 | -80.0–45.0 |  | -3.4 ± 22.8 | -40.0–45.0 | 7.3 | 0.15 | 1.00 |
| Emotion misattribution | -12.0 ± 15.9 | -46.7–20.0 |  | -6.3 ± 15.0 | -35.0–26.7 | 6.1 | 0.07 | 0.75 |
| Emotion detection | 0.03 ± 14.7 | -41.4–25.9 |  | -3.0 ± 13.5 | -39.3–17.9 | -2.2 | 0.46 | 1.00 |
| Affective memory | 1.8 ± 13.0 | -20.7–35.3 |  | -3.8 ± 14.3 | -30.7–32.7 | -4.5 | 0.13 | 1.00 |
| *Hot cognition II: social cognition* | | |  |  |  |  |  |  |
| Guilt ratings | 4.2 ± 0.7 | 2.9–5.9 |  | 4.2 ± 0.6 | 3.2–5.5 | -0.01 | 0.93 | 1.00 |
| Shame ratings | 4.4 ± 0.8 | 2.7–5.8 |  | 4.4 ± 0.7 | 3.2–6.3 | 0.03 | 0.84 | 1.00 |
| Information sampling | 36.5 ± 17.7 | -21.9–71.9 |  | 39.9 ± 19 | -9.4–78.1 | 3.1 | 0.44 | 1.00 |
| Social interpretation bias | 9.9 ± 23.6 | -50.0–50.0 |  | 3.3 ± 18.9 | -43.8–50.0 | -4.3 | 0.30 | 1.00 |
| *Cold cognition* |  |  |  |  |  |  |  |  |
| Verbal memory | 14.3 ± 3.6 | 6.9–21.9 |  | 15.1 ± 4.5 | 5.5–23.1 | 0.8 | 0.41 | 1.00 |
| Working memory | 11.2 ± 2.6 | 6–16 |  | 12.0 ± 3.0 | 6–18 | 0.8 | 0.15 | 1.00 |
| Reaction time | 285 ± 57.6 | 205.4–466.7 |  | 270.1 ± 62.6 | 200.5–465.7 | -17.2 | 0.17 | 1.00 |
| **Figure S3.2.** Group differences between patients with first episode-depression and recurrent depression. The group differences were assessed with linear regression models with cognitive task score as the dependent variable and depression history (first-episode depression was coded as 0 and recurrent depression as 1), age and sex as independent variables. Uncorrected *p*-values and *p*-values corrected for 11 tests using the Bonferroni-Holm method are shown. | | | | | | | | |

Table S3.2 shows differences in cognitive performance between patients with first-episode depression and recurrent depression. We detected no statistically significant differences in performance on any of the cognitive tasks.

**4. Correlation between cognitive scores and clinical symptoms**

Figure S4.1 shows Spearman’s ranked order correlations between clinical depression symptoms indexed with Hamilton Depressive Ratings Scale-6 (HDRS_6_) and Hamilton Depressive Ratings Scale-17 (HDRS_17_) and scores on cognitive tasks for all depressed patients (N = 92) and the three cognitive profile clusters.

**Figure S4.1 Correlation between cognitive scores and clinical symptoms**

After correction for 11 tests, none of the *p*-values were statistically significant for either HDRS_6_ scores (all *p_corrected_* > 0.42) or HDRS_17_ (all *p_corrected_* > 0.11).

**5. Cognitive task scores across groups**

Table S5.1. shows the average scores on cognitive tasks for the all healthy controls (N = 103) and patients (N = 92).

**Table S5.1. Performance on cognitive tasks**

|  | Healthy controls (N = 103) | |  | Depressed patients (N = 92) | |
| --- | --- | --- | --- | --- | --- |
|  | Mean ± SD | Range |  | Mean ± SD | Range |
| *Hot cognition: affective biases* | | | | |  |
| Emotion recognition | 6.7 ± 26.3 | -55.0–90.0 |  | -6.1 ± 23.5 | -80.0–45.0 |
| Emotion misattribution | -0.05 ± 17.9 | -35.0–61.7 |  | -8.8 ± 15.6 | -46.7–26.7 |
| Emotion detection | 7.1 ± 13.0 | -40.2–36.9 |  | -1.7 ± 14.0 | -41.4–25.9 |
| Affective memory | 0.2 ± 12.5 | -28.0–29.3 |  | -1.2 ± 13.9 | -30.7–35.3 |
| *Hot cognition: social cognition* | | | |  |  |
| Guilt ratings | 3.7 ± 0.5 | 1.8–5.1 |  | 4.2 ± 0.6 | 2.9–5.9 |
| Shame ratings | 3.9 ± 0.6 | 2.1–5.5 |  | 4.4 ± 0.8 | 2.7–6.3 |
| Information sampling | 74.1 ± 16.9 | 0.0–100.0 |  | 77.3 ± 18.8 | 0.0–100.0 |
| Social interpretation bias | 10.6 ± 22.3 | -50.0–75.0 |  | 6.1 ± 21.2 | -50.0–50.0 |
| *Cold cognition* | | |  |  |  |
| Verbal memory | 17.0 ± 3.7 | 8.5–23.1 |  | 14.7 ± 4.1 | 5.5–23.1 |
| Working memory | 13.6 ± 3.2 | 5.0–20.0 |  | 11.6 ± 2.8 | 6.0–18.0 |
| Reaction time | 238.2 ± 41.8 | 191.0–439.0 |  | 276.8 ± 60.5 | 200.5–466.7 |

Table S5.2. shows the average scores on cognitive tasks for the three cognitive profiles clusters.

| **Table S5.2. Group differences** | | | |  |  |  |  |  |  |
| --- | --- | --- | --- | --- | --- | --- | --- | --- | --- |
|  |  | Cluster A (N = 38) | |  | Cluster B (N = 28) | |  | Cluster C (N = 26) | |
|  |  | Mean ± SD | Range |  | Mean ± SD | Range |  | Mean ± SD | Range |
| *Hot cognition: affective biases* | | | |  |  |  |  |  |  |
| Emotion recognition |  | -20.7 ± 16.9 | -80.0–15.0 |  | 18.8 ± 14.9 | -10.0–45.0 |  | -11.7 ± 17.4 | -40.0–20.0 |
| Emotion misattribution |  | -17.6 ± 11.6 | -46.7–3.3 |  | 7.9 ± 10.2 | -13.3–26.7 |  | -14.0 ± 10.7 | -35.0–6.7 |
| Emotion detection |  | 0.5 ± 12.6 | -32.1–19.6 |  | 1.5 ± 10.4 | -17.9–17.9 |  | -8.2 ± 17.3 | -41.4–25.9 |
| Affective memory |  | 1.6 ± 14.8 | -24.0–32.7 |  | -4.9 ± 14.9 | -30.7–35.3 |  | -1.3 ± 10.9 | -18–19.3 |
| *Hot cognition: social cognition* | | | |  |  |  |  |  |  |
| Guilt ratings |  | 3.9 ± 0.4 | 3.1–4.9 |  | 4.0 ± 0.5 | 2.9–5.0 |  | 5.0 ± 0.4 | 4.0–5.9 |
| Shame ratings |  | 4.1 ± 0.6 | 2.7–5.0 |  | 4.0 ± 0.5 | 2.9–5.1 |  | 5.3 ± 0.5 | 4.1–6.3 |
| Information sampling |  | 38.5 ± 18.1 | -21.9–78.1 |  | 36.7 ± 18.3 | 0.0–68.8 |  | 40.3 ± 19.6 | -9.4–71.9 |
| Social interpretation bias |  | 4.0 ± 22.2 | -50.0–37.5 |  | 10.3 ± 16.9 | -18.8–50 |  | 4.5 ± 24.0 | -37.5–50.0 |
| *Cold cognition* |  |  |  |  |  |  |  |  |  |
| Verbal memory |  | 16.8 ± 3.2 | 9.8–23.1 |  | 14.3 ± 3.6 | 6.2–23.0 |  | 12.3 ± 4.4 | 5.5–23.1 |
| Working memory |  | 12.5 ± 2.8 | 8.0–18.0 |  | 11.6 ± 2.5 | 6.0–15.0 |  | 10.5 ± 2.8 | 6.0–16.0 |
| Reaction time |  | 267.4 ± 49.5 | 200.5–454.7 |  | 278.8 ± 57.8 | 219.3–436.9 |  | 288.9 ± 76.9 | 205.4–466.7 |

**6. Effect of IQ and education on group difference estimates**

Table S6.1. shows IQ scores indexed with the Reynolds Intellectual Screening Test (RIST) and education levels indexed with the Online Stimulant and Family History Assessment Module (OS-FHAM) questionnaire for the depressed patients (N = 92) and healthy controls (N = 103). Table S6.2. shows group difference estimates after correction with IQ and education respectively.

**Table S6.1. Education and IQ**

|  | Depressed patients (n = 92) | Healthy controls (n = 103) | *p*-value |
| --- | --- | --- | --- |
| IQ score | 102.9 ± 8.4 (86–124)^a^ | 110.2 ± 7.0 (93–129) | <0.001 |
| Education | 16.4 ± 1.3 (11–17)^c^ | 14.9 ± 2.2 (8–17)^b^ | <0.001 |
| **Table S6.1.** The table shows IQ indexed with the Reynolds Intellectual Screening Test (RIST) and education score indexed with the Online Stimulant and Family History Assessment Module (OS-FHAM) as completed number of school years added to an education score between 1 (no vocational degree) and 5 (> 4 years of higher learning at university level). Group differences were assessed with an independent *t-*test. ^a^N = 87, ^b^N = 102, and ^c^N = 74 due to missing data. | | | |

**Table S6.2.** **Group differences on cognitive performance corrected for IQ and education**

|  | Corrected for IQ | |  | Corrected for education | |
| --- | --- | --- | --- | --- | --- |
|  | *β* | *p* |  | *β* | *p* |
| Emotion processing |  |  |  |  |  |
| Recognition bias | -12.76 | 0.002 |  | -10.85 | 0.01 |
| Misattribution bias | -7.42 | 0.01 |  | -7.39 | 0.01 |
| Detection bias | -8.419 | < 0.001 |  | -9.76 | < 0.001 |
| Affective memory | -1.326 | 0.54 |  | -2.08 | 0.35 |
| Social cognition |  |  |  |  |  |
| Guilt ratings | 0.49 | < 0.001 |  | 0.48 | < 0.001 |
| Shame ratings | 0.54 | < 0.001 |  | 0.54 | < 0.001 |
| Information sampling | 2.029 | 0.50 |  | 1.82 | 0.56 |
| Social interpretation bias | -2.12 | 0.58 |  | -0.03 | 1.00 |
| Cold cognition |  |  |  |  |  |
| Verbal memory | -1.60 | 0.009 |  | -1.84 | 0.004 |
| Working memory | -0.81 | 0.07 |  | -1.20 | 0.02 |
| Reaction time | 23.5194 | 0.01 |  | 28.29 | 0.005 |
| **Table S7.2.** Group difference between patients (N = 92) and healthy controls (N = 103) on primary cognitive outcomes after correction for IQ and correction for education. Note *p*-values are reported uncorrected; all models were corrected for age and sex. | | | | | |
